# Synthesis and Potential Applications of Lipid Nanoparticles in Medicine

**DOI:** 10.3390/ma15020682

**Published:** 2022-01-17

**Authors:** Ewelina Musielak, Agnieszka Feliczak-Guzik, Izabela Nowak

**Affiliations:** Faculty of Chemistry, Adam Mickiewicz University in Poznań, Uniwersytetu Poznańskiego 8, 61-614 Poznań, Poland; ewelina.musielak@amu.edu.pl (E.M.); agaguzik@amu.edu.pl (A.F.-G.)

**Keywords:** nanotechnology, nanostructured materials, lipid nanoparticles, biocompatible pharmaceutical carriers

## Abstract

Currently, carriers of active ingredients in the form of particles of a size measured in nanometers are the focus of interest of research centers worldwide. So far, submicrometer emulsions, liposomes, as well as microspheres, and nanospheres made of biodegradable polymers have been used in medicine. Recent studies show particular interest in nanoparticles based on lipids, and at the present time, are even referred to as the “era of lipid carriers”. With the passage of time, lipid nanoparticles of the so-called first and second generation, SLN (Solid Lipid Nanoparticles) and nanostructured lipid carriers and NLC (Nanostructured Lipid Carriers), respectively, turned out to be an alternative for all imperfections of earlier carriers. These carriers are characterized by a number of beneficial functional properties, including, among others, structure based on lipids well tolerated by the human body, high stability, and ability to carry hydro- and lipophilic compounds. Additionally, these carriers can enhance the distribution of the drug in the target organ and alter the pharmacokinetic properties of the drug carriers to enhance the medical effect and minimize adverse side effects. This work is focused on the current review of the state-of-the-art related to the synthesis and applications of popular nanoparticles in medicine, with a focus on their use, e.g., in COVID-19 vaccines.

## 1. Introduction

The intensive development of nanotechnology and continuous work on the miniaturization of technological objects has resulted in the possibility of obtaining structures with sizes not exceeding 100 nm. Reducing the particle size of materials to the nanometer scale makes it possible to increase their total surface area by several orders of magnitude. Such objects are called nanoparticles, and methods for their production are the subject of nanotechnology. The origin of the term “nano” is derived from the Greek word “nanos”, which means “dwarf” [1]. Nanoparticles are very attractive research topics due to their potential applications in biotechnology, pharmaceuticals, and optics, but most importantly in medicine [2]. The number of reports and articles on nanoparticles is significant and is expected to increase in the next few years.

Nanoparticles are defined as particles with at least one spatial dimension, not exceeding 100 nm [3]. According to their origin, they can be divided into two types. Natural nanoparticles are commonly found in the natural environment, mostly in volcanic smoke. Natural nanoparticles are produced by the erosion of geological materials and the degradation of biological materials, mainly vegetable residues, and can be produced by combustion processes. The second type is engineered nanoparticles (designed) with a small size that show a tendency towards fast aggregation. Engineered nanoparticles include fullerenes, carbon nanotubes, quantum dots, and nanofibers [4].

The use of nanoscale materials provides great freedom to modify basic parameters such as immunogenicity, diffusivity, solubility, half-life in the bloodstream, and release rate of the active substance. Over the past two decades, several nanoparticle-based therapeutic agents have been developed for the treatment of diabetes, asthma, allergies, pain, infections, and others [5].

The role of nanomedicine is to provide more efficient tools to prevent and treat various diseases through the interaction of nanomaterials with biological molecules. Nanomedicine-based strategies have opened new horizons for biomedical engineers and clinicians in the prevention, diagnosis, and treatment of serious diseases. With nanotechnology applied to medicine, significant improvements have been registered in drug delivery systems, protein detection, cancer treatment, medical imaging and diagnostic platforms, implantable materials, and tissue regeneration strategies, among others [6,7]. It is important to mention that various nanosystems are already being used with a positive impact on human health and that even more medical projects are in development.

Among the nanoparticles described above, in recent years, lipid nanoparticles have received the most attention from researchers because drug delivery systems based on them, because of their lipophilicity, show the ability to overcome difficult physiological barriers, such as blood-brain, even without surface modification. Other very important beneficial properties offered by lipid nanoparticles are high skin compatibility, higher penetration through the stratum corneum, high mechanical and chemical versatility, biodegradability, and protection of the active ingredient from degradation processes induced by external factors. The lipid nanoparticles show low toxicity, have good conformity with biological substances, and most of the preparation methods can be easily scaled up. However, additional studies are necessary on the interaction and boundary of active substances with lipids and surfactants/emulsifiers and on the mechanisms of diffusion and cellular uptake to achieve optimal working conditions. Another important hot topic to be elaborated on is the problems related to the encapsulation of hydrophilic compounds. The important concepts to be addressed while encapsulating hydrophilic compounds include the solubility of the molecule, the partitioning of the active substance and lipid, and the mass transport phenomena. The solubility of the bioactive compound or drug must always be analyzed not only in the different phases of the delivery system but also in the surrounding application matrix.

## 2. Characteristics of Nanoparticles

**Figure 1:**
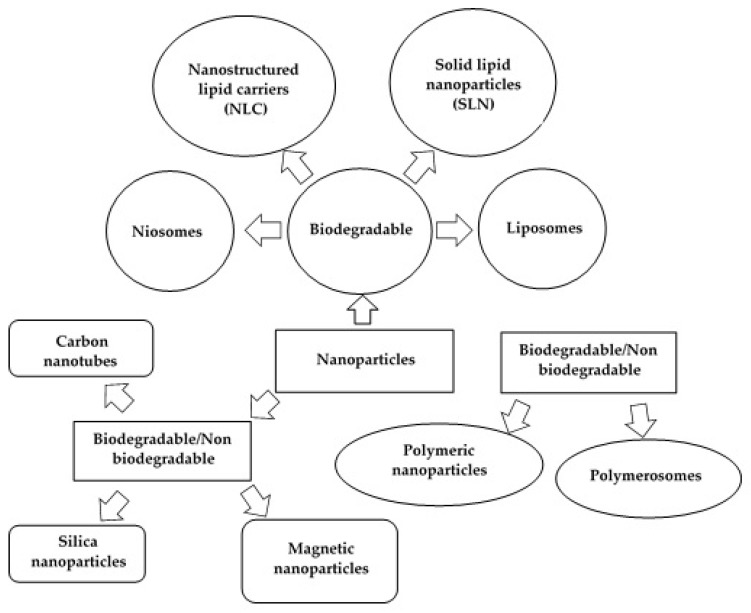
Schematic diagram showing different types of nanoparticles (modified from [1]; original paper under the terms of the Creative Commons CC BY-NC 3.0).

Nanoparticles as drug carriers are gaining popularity, mainly due to their small size and unique properties that make them attractive in many fields of science. Such carriers offer a number of important benefits, including protection of the active ingredient from moisture, physiological pH, and enzyme activity. Of the group of nanocarriers shown in Figure 1, liposomes, niosomes [1], and lipid matrix nanoparticles, including solid lipid nanoparticles (SLN) and nanostructured lipid carriers (NLCs) [2,8,9], have received particular attention.

Currently, the most popular carriers for active ingredients are solid lipid nanoparticles (SLNs, first-generation lipid nanoparticles) and nanostructured lipid carriers (NLCs, second-generation lipid nanoparticles) [10]. Second-generation nanoparticles were synthesized to improve the properties of the first-generation particles. The most significant difference between these two types of systems is the incorporation of liquid lipids into the structure of NLC-type materials [11].

## 3. Liposomes

Liposomes are spherical vesicles, 0.01–1 µm in size, composed of 1 or more phospholipid bilayers formed from cholesterol and other natural phospholipids, separated by spaces containing water [12,13]. Typically, compounds that form the lipid bilayer are phosphatidylcholine (PC), phosphatidylglycerol (PG), dipalmitoylphosphatidylcholine (DPPC), distearoylphosphatidylcholine (DSPC), cholesterol (CH), stearylamine, dicetyl phosphate, or mixtures thereof [14,15].

The size of the vesicle is an important parameter in estimating the half-life of liposomes in the bloodstream. Both the size of the liposome and the number of bilayers in it have a great influence on drug encapsulation [14]. According to the size, the number of bilayers, and the way liposomes are obtained, they can be divided into [16]:

- multilamellar vesicles MLV;
- unilamellar vesicles UV;
- small unilamellar vesicles SUV;
- large unilamellar vesicles LUV;
- multivesicular vesicle MVV.

The size of a small unilamellar vesicle SUV is in the range of 0.02–0.1 μm (20–100 nm), while the size of LUV vesicles is >0.1 μm (100 nm) and MLV vesicles >0.5 μm (500 nm). Multilamellar vesicle structures are >1 μm (1000 nm) in size. For medical applications of liposomes related to drug delivery, the desired size is in the range of 0.05–0.2 μm (50–200 nm) [16].

Unilamellar liposomes are composed of a single phospholipid bilayer surrounding an aqueous solution, whereas multilamellar liposomes form an onion-shaped structure. Classically, in the case of multilamellar liposomes, several monolayer vesicles of smaller size are formed on the inner side of the second layer, forming a multilamellar structure of concentric lipid globules separated by layers of water [17].

Liposomes occur naturally in living organisms, for example, in blood, but can also be produced for the pharmaceutical and cosmetic industries [13]. They are classified as vesicular delivery systems that can carry hydrophilic or hydrophobic substances. They are small spherical particles, have high EE% (encapsulation efficiency) of the drug, and contain an inner aqueous core and an outer double lipid layer [18].

The properties of liposomes depend on their composition and size, method of production, and quantity of the surface charge. By selecting appropriate components of the bilayer, it is possible to adjust its stiffness, fluidity, and surface charge. For example, the use of phosphatidylcholine from natural sources (eggs or soy) makes the bilayer less rigid and more permeable. In contrast, the use of phospholipids with long acyl chains makes the resulting bilayer rigid and impermeable [14]. Liposomes, due to their unique properties, can be used in medicine. Their clinical applications include cancer treatment and antimicrobial and gene therapies [19,20,21].

Liposomes exhibit a number of special biological features, including (specific) interactions of liposomes with biological membranes. The main advantages of liposomes are biocompatibility, biodegradability, and non-immunogenicity. They are flexible, non-toxic, and increase of efficacy and therapeutic index of a drug. Liposomes increase drug/molecule stability as a result of encapsulation. The disadvantages of liposomes are low solubility and stability, short half-life, and high production cost. There is a possibility of phospholipid oxidation and hydrolysis-like reaction [14].

### 3.1. Methods for Obtaining Liposomes

There are several basic strategies involved in the synthesis of liposomes for various applications. They include four basic steps:

- separation of the lipids from the organic solvent;
- dispersion of the lipids in an aqueous medium;
- purification of the resulting liposome;
- analysis of the final product.

The most variable step in the process of obtaining liposomes is the dispersion of lipids in an aqueous medium [12]. The most common methods for obtaining liposomes are summarized in Table 1.

### 3.2. Applications of Liposomes

Liposomes are mainly used for drug delivery. One method to improve the effectiveness of treatment is to use active substances encapsulated in carriers and administer them at concentrations that ensure delivery to the target sites without damaging healthy tissues. Liposomal drug carriers can provide great benefits compared to traditional drug substances. They protect the encapsulated drug from degradation and premature metabolization during release into the human body [14]. Encapsulation of active substances in liposomes can be achieved by two methods:

- passively—the active substance is encapsulated during liposome formation;
- actively—the active substance is encapsulated after the liposomes are formed.

Hydrophobic drugs, for example, amphotericin B, taxol, or annamycin, can be directly combined with liposomes during vesicle formation, and their absorption and encapsulation depend on drug-lipid interactions. It is possible to achieve 100% drug encapsulation efficiency, but it depends on the solubility of the drug in the liposome membrane [12]. Liposomes are used to deliver drugs approved by the Food and Drug Administration (FDA), like the anthracyclines Doxil (doxorubicin) and DaunoXome (daunorubicin), for the treatment of ovarian cancer, breast cancer, multiple myeloma, and sarcomas [26].

Numerous studies have led to “second generation liposomes”, i.e., the structures with modified surfaces. They are obtained by using several types of particles, such as glycolipids or sialic acid (N-acetylneuraminic acid), as well as modified and unmodified dextrans [14]. Liposomes, due to the high risk of “uptake” by the reticuloendothelial system, are coated with a synthetic polymer, polyethylene glycol (PEG). The use of PEG prolongs the period of liposome circulation in blood [21,27,28]. Unfortunately, limitations to the use of liposomes include short lifespan, low encapsulation efficiency (mainly hydrophilic drugs), and rapid “uptake” [29]. It has been confirmed that drug encapsulation in liposomes or solid lipid nanoparticles increases their delivery to the brain; for some of them, this is the only chance to cross the blood-brain barrier. Liposomes are also used in gene therapy as non-viral vectors [30]. Currently, only positively charged liposomes are used for this purpose because the cationic surface allows complexes to form with the negatively charged DNA structure. The purpose of using liposomes as nonviral vectors is to deliver the unmodified gene to the target cell. Liposome vesicles have found wide application in the delivery of proteins and peptides into the cytoplasm of dendritic cells by endocytosis [31]. The main advantage of liposomes as gene carriers over non-viral vectors [30] is that they do not contain proteins, which eliminates a possible immune response. On the other hand, their disadvantage is that they can accumulate in a living organism and thus fail to perform their task. However, this disadvantage can be overcome by coating them with neutral or cationic phospholipids, as proposed by Miller and his team at Imperial College, London [32].

## 4. Niosomes

Niosomes are nanocarriers formed by the self-organization of nonionic surfactants in an aqueous environment, resulting in closed bilayer structures [33]. They are composed of a nonionic surfactant, cholesterol or its derivatives, and a charged molecule, surrounded by aqueous spaces, see Table 2.

Niosomes are classified based on their size or number of bilayers:

- small unilamellar vesicles (SUV);
- large unilamellar vesicles (LUV);
- multilamellar vesicles (MLV) [50].

The particle size of nonionic vesicles is in the sub-micron size range. For SUV vesicles, their size is 10–100 nm, while the size of LUV vesicles is in the range of 100–3000 nm and MLVs are larger than 5 µm [33]. Niosomes have a unique structure, and therefore it is possible to encapsulate hydrophilic and hydrophobic materials in the particle core and in the bilayer [51,52].

**Figure 2:**
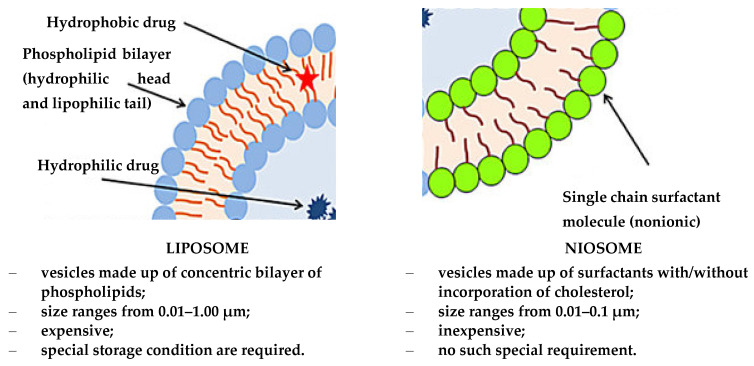
Schematic of the structure of the liposome and niosome (modified from [54]; original paper under the terms of the Creative Commons CC BY-NC 3.0).

The introduction of cholesterol during the synthesis of niosomes improves the rigidity of the bilayer. This structure protects drug molecules from premature degradation and inactivation due to adverse immunological and pharmacological effects [53]. A comparison between liposomes and niosomes is shown in Figure 2.

### 4.1. Methods of Obtaining Niosomes

The most important methods for obtaining niosomes are included in Table 3.

### 4.2. Applications of Niosomes

Niosomes are promising carriers for many pharmaceuticals and may be used in diagnostics. Due to their non-ionic nature, they provide high biocompatibility and low toxicity [33].

Initially, niosomes were synthesized for cosmetic purposes, but over time, they have been proposed for use in the pharmaceutical industry for potential drug delivery. Studies of niosomes such as unilamellar or multilamellar nanoparticles are concerned with determining optimal drug encapsulation methods and the kinetics of loading and release of the active ingredient. The ability of niosomes to improve the bioavailability and efficacy of drugs has attracted the attention of researchers due to their potential use in the treatment of severe inflammation and various types of cancer, among others [33].

When used as novel drug delivery systems, niosomes counteract the poor penetration of chemotherapeutics into cancer cells and offset the side effects of chemotherapeutics on healthy cells. They are used in the treatment of melanoma, lung cancer, breast cancer, and ovarian cancer [59], and they are also effective carriers of antibiotics and anti-inflammatory drugs. In addition, niosomes have been synthesized to improve poor drug penetration through the skin and poor drug retention in the skin. Studies conducted for rifampicin, a broad-spectrum antibiotic, have shown that niosomes used as carriers for this drug provide continuous and prolonged release [61].

Niosomes are also being tested as carriers of anti-inflammatory drugs, especially nonsteroidal anti-inflammatory drugs (NSAIDs), which are known to cause adverse side effects such as mucosal irritation. The topical application of NSAID-loaded niosomes improves drug permeability. Studies have been carried out on niosomes containing ammonium aglycyrrhizinate (AG), using several surfactants and cholesterol at different concentrations [62]. The drug entrapment efficiency, lipid anisotropy, cytotoxicity of this active ingredient delivery system, and its skin tolerance were evaluated. AG-loaded niosomes showed no toxicity, and their use improved the anti-inflammatory effect of the drug. In addition, enhancement of the anti-inflammatory effect of drugs delivered by niosomes on chemically induced skin erythema in humans has been reported. These nanoparticles are also used to deliver antiviral drugs, mainly against HIV [61].

## 5. Lipid Nanoparticles

Lipid matrix nanoparticles (LNPs), which are used as nanocarriers, were first synthesized in the early 1990s [63]. Their synthesis was a breakthrough in the search for a new generation of nanocarriers. Due to the wide possibilities of their surface modifications, they can be used to increase drug bioavailability, enable controlled release of active ingredients, improve intracellular permeability, and enable control of drug delivery to target sites [1].

Lipid-based nanoparticles are colloidal particles consisting of a lipid matrix stabilized with surfactants. They are stable at room temperature [64], their size ranges from 40 nm to 1000 nm, and they are called lipid colloidal carriers [65,66]. Typically, LNP carriers consist of solid lipids, surfactants, cosurfactants (if necessary), and active ingredients. The composition of the lipids is crucial [67]. The most commonly used lipids are fatty acid esters, fatty alcohols, acylglycerols, and mixtures of acylglycerol esters. Examples of lipids used in LNP synthesis are summarized in Table 4.

### 5.1. Solid Lipid Nanoparticles (SLNs)

**Figure 3:**
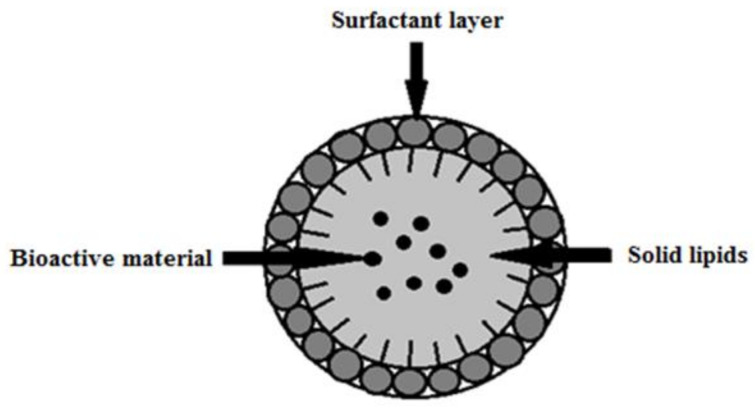
Structure of a solid lipid nanoparticle (SLN) stabilized by surfactant coating/layer (modified from [1]; original paper under the terms of the Creative Commons CC BY-NC 3.0).

Solid lipid nanoparticles (SLNs) are colloidal carriers that were first synthesized in 1991 as an alternative to liposome, emulsion, and polymeric micro- and nanoparticle-based systems [68]. SLNs combine the advantages of traditional colloidal carriers and avoid some of their major drawbacks [69]. They are stable at room temperature and at human body temperature [70,71]. SLNs usually have a spherical shape and a diameter in the range of 50–1000 nm. Solid lipid nanoparticles consist of a core of solid lipids in which a bioactive component is embedded, and this structure is stabilized by a surfactant coating (Figure 3).

Depending on the structure, lipids are mainly divided into fatty acids, fatty esters, fatty alcohols, triacylglycerols, and partial glycerides. Ionic and nonionic polymers (Pluronic^®^, such as F68 and F127 (e.g. Sigma-Aldrich Sp. z.o.o (Poznań, Poland)), surfactants, and organic salts are used as emulsifiers [1]. The most attractive features of SLNs are:

- high physical stability;
- the possibility of large-scale synthesis;
- biocompatibility;
- lack of biotoxicity;
- increased stability of the active ingredient;
- low risk of chronic or acute intoxication;
- no need for organic solvents in synthesis [71];
- the ability of controlled release of active ingredient [72];
- ability to block UV radiation (UV blocking is characteristic of some lipids);
- the ability to act as occlusive compounds;
- the ability to protect unstable compounds from chemical degradation [73].

Potential disadvantages associated with SLNs are limited drug loading capacity, the potential for drug leakage (uncontrolled release), and crystallization during formulation storage [1].

### 5.2. Nanostructured Lipid Carriers (NLCs)

Nanostructured lipid carriers are the second generation of solid lipid nanoparticles that have been designed to eliminate the SLN limitations associated with having to use only solid lipids for their synthesis. Müller et al. [74] have shown that it is possible to overcome this limitation by adding liquid lipids that alter the crystal structure of the lipid matrix [74,75]. NLCs offer a number of additional benefits, such as greater drug loading capability and higher physicochemical stability attributed to a mixture of solid and liquid lipids [76,77]. Nanostructured lipid carriers are characterized by a partially crystallized lipid matrix with a poorly ordered structure, which significantly reduces the risk of drug leakage (uncontrolled release) during storage [78].

The technology for obtaining NLCs is identical to that of SLNs. Nanostructured lipid carriers can be produced by the HPH (High-Pressure Homogenization) method, and this process can result in particle dispersions with solid content in the range of 30–80 wt.% [69].

**Figure 4:**
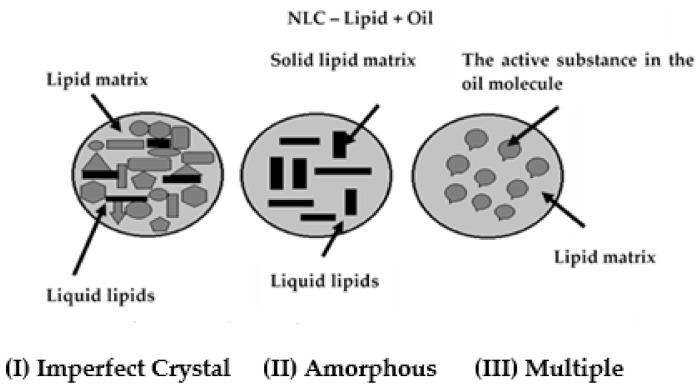
Structure types of nanostructured lipid carriers: (**I**) imperfect crystal, (**II**) amorphous, (**III**) multiple (modified from [2]; original paper under the terms of the Creative Commons CC BY).

Depending on the composition of the lipid-oil mixture, three types of NLC structures can be distinguished (Figure 4) [2]:

- imperfect crystal structure;
- amorphous structure;
- multiple structure.

NLCs cannot be used to encapsulate active ingredients and drugs with a hydrophilic character. In contrast to SLNs, the NLC matrix consists not only of solid lipids but also contains liquid lipids (oils), water, and surfactants. SLN is mixed with oils in ratios ranging from 70.0:30.0 to 99.9:0.1. Higher amounts of oils can be used in the NLC multilayer structure. The entire system is stabilized with surfactants (0.5–5.0% by weight). The compounds most commonly used for NLC synthesis are [1,2]:

❖ **Solid lipids**
  ➢ glyceryl behenate (e.g., Compritol^®^ 888 ATO, Gattefosse (Saint-Priest, France));
  ➢ stearic acid;
  ➢ glyceryl monostearate (GMS);
  ➢ cetyl palmitate;
  ➢ glyceryl palmitostearate (e.g., Precirol^®^ ATO 5 Gattefosse (Saint-Priest, France));.
❖ **Liquid lipids**
  ➢ soybean oil;
  ➢ oleic acid (OA);
  ➢ isopropyl myristate;
  ➢ α-tocopherol/vitamin E;
  ➢ squalene.
❖ **Surfactants**
  ➢ polyoxyethylene (20) sorbitan monolaurate (e.g., Tween^®^ 20 Sigma-Aldrich Sp. z.o.o (Poznań, Poland));
  ➢ polyoxyethylene (80) sorbitan monooleate (e.g., Tween^®^ 80 Sigma-Aldrich Sp. z.o.o (Poznań, Poland));
  ➢ polyvinyl alcohol (PVA);
  ➢ ecithin;
  ➢ Solutol^®^ HS 15; (e.g., Sigma-Aldrich Sp. z.o.o (Poznań, Poland));
  ➢ polyoxyl castor oil.

The NLC core with an imperfect crystal structure (Figure 4I) consists of a mixture of solid and liquid lipids. The structural differences between these two components contribute to the modification of the packed structure of the solid lipids. Crystallization processes of the imperfect matrix result in a disordered structure, offering free space that can be occupied by the active component [1,2,74].

NLCs with an amorphous structure have a core of an amorphous matrix formed by mixing large amounts of oil with solid lipids. Usually, drugs are much more soluble in liquid than in solid lipids, which results in the production of NLCs with high liquid lipid content. In the fabrication of NLCs with an amorphous structure, the liquid lipid particles (nanoemulsions) are cooled to room temperature to initiate crystallization and solid particle formation. In the amorphous type of structure, the oil concentration is high, and after cooling, crevices form where solid and liquid lipids can mix **(**Figure 4II). NLCs are mostly found in the amorphous state [79]. The third type of NLC with multiple structures is also called the amorphous type. In this method of NLC preparation, lipids are mixed to prevent crystallization. In NLC with multiple structure, the lipid matrix remains solid but in an amorphous state (Figure 4III) [80]. Upon cooling, phase separation occurs, and oil nanoparticles precipitate. The active ingredient is dissolved in the liquid lipid so that it is protected from the aqueous environment [69,81].

### 5.3. Methods of Obtaining Nanoparticles

The content of this section is limited to a discussion of methods for the synthesis of lipid nanoparticles. The main methods for this synthesis are high-pressure homogenization (HPH) [70] and the microemulsion method [82]. Other cheaper and easier methods have also been proposed, such as dispersion using a high-speed stirrer or ultrasound, solvent-based emulsification/evaporation technique, or membrane squeezing method [71].

#### 5.3.1. High-Pressure Homogenization (HPH)

High-pressure homogenization is the most efficient method for lipid nanoparticle synthesis and has been used for many years for microemulsion production and large-scale production of lipid nanoparticles of both SLN and NLC types [8,80]. The greatest advantages of this technique are the very short production time and the easy transfer of production from laboratory scale to large-scale production [83].

**Figure 5:**
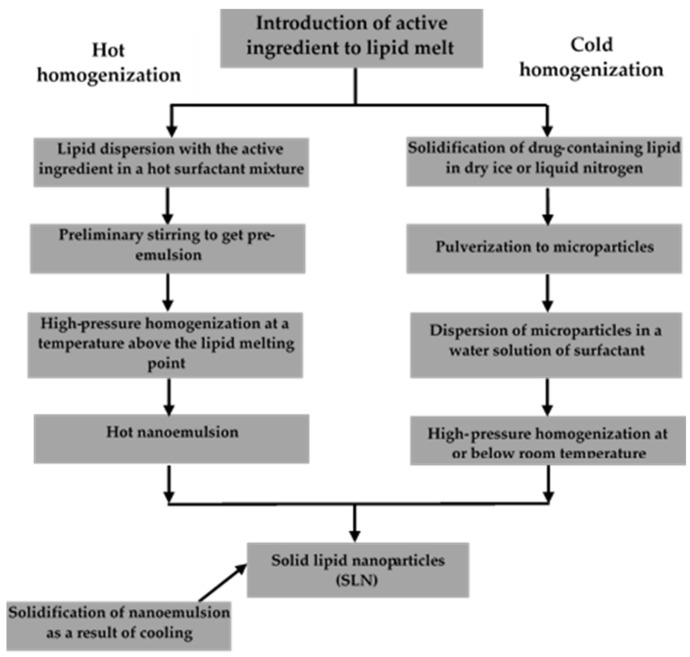
Hot and cold homogenization scheme (modified from [1]; original paper under the terms of the Creative Commons CC BY-NC 3.0).

Despite its many advantages, high-pressure homogenization is an energy-intensive process and causes an increase in sample temperature, which is not beneficial for heat-sensitive compounds [80]. In high-pressure homogenizers, a mixture of lipids, water, surfactant, and the active ingredient is forced through very small diameter tubes (usually a few microns) at pressures ranging from 100 bar to 2000 bar. Over short distances, the liquid can reach velocities of more than 1000 km/h. As a result of high cavitation and shear forces, the liquid lipid particles are fragmented to a submicrometer size. This method can achieve lipid nanodispersion even at lipid concentrations of up to 40% by weight [84]. There are two types of high-pressure homogenization: hot and cold. Schemes of both processes are shown in Figure 5. In both methods, in the initial stage, the active ingredient is introduced into the lipid particles by dissolution or dispersion in molten lipid [85].

**Hot Homogenization HPH**

Hot homogenization (Figure 5) is performed at temperatures above the melting point of lipids and is often considered emulsion homogenization. A high-speed stirrer is used to obtain a pre-emulsion containing the active ingredient, the molten lipid, and the aqueous phase of the emulsifier (5–10 °C above the melting point of the lipid). The quality of the final homogenization product depends largely on the quality of the pre-emulsion, which should be made of particles of a few micrometers in size. Too high a temperature can lead to degradation of the active ingredient and/or the carrier. The homogenization process can be repeated several times. Homogenization under increased pressure can lead to an increase in the emulsion temperature of up to approximately 10 °C for every 500 bar of pressure applied. Generally, satisfactory results are obtained after 3 to 5 cycles of homogenization at 500 to 1500 bar pressure. Increasing the pressure or the number of cycles increases the size of the lipid nanoparticles as a result of coalescence occurring due to their high kinetic energy. The final product of hot homogenization is a nanoemulsion in the liquid phase. Particulate solids are obtained after the nanoemulsion is cooled to room temperature or below. Lipid crystallization can take a very long time (up to several months) due to the small size of the particles and the presence of emulsifiers [85].

**Cold Homogenization HPH**

This process is ideal for highly sensitive hydrophilic compounds [86]. Cold homogenization (Figure 5) is performed with solid lipids. In this process, proper temperature control is particularly important to maintain the solid lipids since the temperature can increase significantly during the homogenization process. The cold homogenization method was developed to overcome the three main disadvantages of hot homogenization [87]:

- high temperature-induced degradation of the active ingredient;
- the complexity of the nanoemulsion crystallization step leading to the modification of lipid nanoparticles;
- the degradation of the active ingredient in the aqueous phase during the homogenization process.

The first stage of cold homogenization is the same as that of the hot homogenization process and involves the solubilization or dispersion of the active ingredient in the molten lipid. In the next step, the molten lipid containing the active ingredient is immediately cooled with dry ice or liquid nitrogen. The efficiency of homogenization of the active ingredient inside the lipid carrier is favored by a significant drop in temperature. The solid lipid containing the active substance is crushed in a mortar into 50–100 microns particles. The low temperatures of the process enhance the brittleness of the lipids, which promotes their efficient pulverization. The solid lipid microparticles are dispersed in a cooled emulsifier solution. The suspension thus obtained is homogenized under increased pressure at room temperature or below. Cold homogenization allows obtaining lipid particles larger than those obtained in the case of hot homogenization. At the same time, it eliminates the risk of adverse effects of high temperatures without taking into account the high temperature of the first step of introducing the active substance into the molten lipid [1,83,85].

#### 5.3.2. Microemulsion Method

The widely used microemulsion method to produce lipid nanoparticles has been described for the first time by Gasco et al. [81]. The method involves melting a lipid (or lipid mixture) and heating the aqueous phase (containing surfactant) to the same temperature. To obtain microemulsions containing solid lipids (at room temperature), the manufacturing process should be carried out at temperatures higher than the melting point of the lipid. The microemulsion is prepared by adding an aqueous solution to the lipid phase upon continuous slow stirring. Lipid nanoparticles are obtained by dispersing the microemulsion in cold water (2–10 °C) by continuous stirring. Finally, the system is washed with distilled water, filtered (to remove larger particles), and can be lyophilized to remove excess water [88].

**Figure 6:**
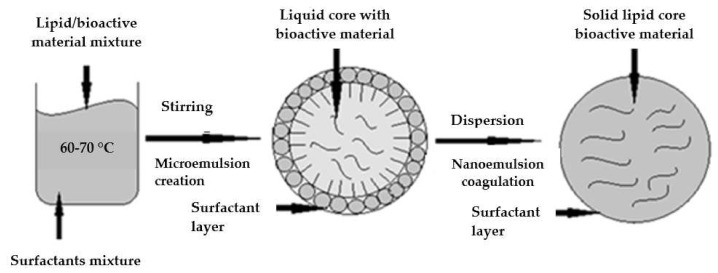
Structures formed by microemulsion fabrication of lipid nanoparticles (modified from [1]; original paper under the terms of the Creative Commons CC BY-NC 3.0).

The most common emulsifiers soy phosphatidylcholine, polysorbate 20, polysorbate 60, while the most commonly used co-emulsifiers are alcohols. The method allows nanoparticles to be obtained under mild temperature conditions. However, the technique has numerous drawbacks, including the need for relatively high concentrations of surfactants and a high dilution of the particle suspension [1,71,89,90]. The scheme of the process is shown in Figure 6.

#### 5.3.3. Solvent-Based Emulsification/Evaporation Technique

The method of obtaining nanodispersions by precipitation of o/w emulsions was first described by Sjörström and Bergenståhl [91]. The technique involves the formation of an oil-in-water emulsion with a partially miscible solvent (of low toxicity), which is then emulsified in the aqueous phase. The whole process is based on the miscibility of water with this type of solvent, which contains active substances. Once formed, the o/w transition emulsion is transferred to water (during continuous mixing), which is the cause of the diffusion of the solvent into the external phase. In this step, the dispersed phase solidifies, and nanoparticles are formed. The solvent can be further removed by evaporation under reduced pressure [92,93]. The main advantages of this technique are versatility, reproducibility, and ease of implementation, as it does not require large energy inputs and does not expose drugs to temperature stress conditions. The disadvantage of the process is the need to purify and thicken the lipid nanoparticle dispersion [93].

#### 5.3.4. Emulsification-Solvent Evaporation

Lipids are dissolved in organic solvents immiscible with water (e.g., cyclohexane or chloroform) and dispersed in the aqueous phase. The lipid solution forms an emulsion in the aqueous surfactant solution after continuous stirring. The organic phase is then evaporated, causing the lipids to precipitate. The disadvantages of this method are the need for organic solvents and an additional evaporation step [85,94].

#### 5.3.5. Sonication/Ultrasound

This is a dispersion-based method and involves melting the lipid matrix (with the drug) at a temperature of 5–10 °C above its melting point. The molten lipid is then dispersed in an aqueous phase containing a surfactant (at the same temperature) upon rapid stirring to form an emulsion. The entire mixture is subjected to ultrasound irradiation to reduce the size of the droplet and gradually cooled to form the nanoparticle dispersion [82]. The most significant advantage of this method is the use of readily available laboratory equipment [73]. Obtaining lipid nanoparticles requires long sonication times, which increases the risk of contaminating the emulsion with metal filings from the probe. Furthermore, the presence of highly polydisperse formulations with a large number of microparticles can be problematic for some lipid nanoparticle-based drug delivery routes [95,96].

#### 5.3.6. Solvent Injection (or Solvent Displacement)

This method involves dissolving the lipid matrix in a solvent mixed with water and rapidly injecting the mixture through a needle into the mixed aqueous phase containing the surfactant. The technique is easy to implement, versatile, and effective in obtaining lipid nanoparticles. Nevertheless, the use of organic solvents is its drawback [97].

#### 5.3.7. Phase Reversal

This method does not require the use of solvents. It involves mixing individual components of the formulation: lipid matrix, drug, water, and surfactant on a magnetic stirrer using 3 temperature cycles (85–60–85–60–85 °C). The mixture is then subjected to a sudden temperature change; that is, it undergoes thermal shock by diluting the mixture with cold distilled water, resulting in the formation of lipid nanoparticles. The advantage of this technique is that there is no need for organic solvents and that heating takes only a short time. However, it is a time-consuming process [88].

#### 5.3.8. Membrane Contractor

In this method, the molten lipid is forced through a porous membrane under increased pressure, while lipid droplets are deposited on the other side of the membrane. The aqueous phase circulates inside the membrane module and removes the lipid droplets. The method is very simple and has no major drawbacks, and the particle size can be controlled using membranes with different pore sizes [98,99].

To summarize up the advantages and disadvantages for the production of SLN/NLC methods are presented in Table 5.

### 5.4. Applications of Lipid Nanoparticles

Nanotechnology is one of the most intensively developing fields of science, mainly because of the great prospects of nanoparticle applications, especially in medicine. Their use as drug carriers is particularly promising in the diagnosis and treatment of various diseases [100]. Lipid-based nanocarriers are of increasing interest, mainly due to their biocompatibility with the human body. Due to their lipophilicity, they are able to overcome physiological barriers, of which the most important is the blood-brain barrier, even without modifying the particle surface. Nanoparticles can be carriers of proteins, peptides, antibodies, and contrast substances [3,101]. The undoubted advantages of nanocarriers are their easy and cheap production methods and the possibility of large-scale production [10,32].

#### 5.4.1. Solid Lipid Nanoparticles (SLNs)

Over the past few years, solid lipid nanoparticles (SLNs) have found applications in the medicine, pharmaceutical, food, and cosmetic industries [71]. Solid lipid nanoparticles are used in the pharmaceutical industry for controlled drug release and to increase the bioavailability of an active ingredient by altering the dissolution rate. SLNs are also used in parenteral therapies (intramuscular, intravenous injections) [72,102], for oral administration [103], rectal [104] and ocular [105].

The increase in the number of people suffering from cardiovascular diseases, including hypertension, cancer, and obesity, has influenced the development of many types of healthy diets based on the intake of dietary supplements. Bioactive substances isolated from animal or plant material are usually perishable, such as carotenoids, which are insoluble in water. The problem faced by manufacturers of specialty foods is the incorporation of lipophilic bioactive ingredients [71]. Solid lipid nanoparticles have proven to be a very good solution.

SLNs have been used successfully as carriers for drugs with protein and polypeptide structures [70,72]. Proteins are natural molecules isolated from oligopeptides which are the basic building blocks of living organisms, while oligopeptides are organic chemical compounds composed of two or more amino acids linked by a peptide bond. Before the use of SLNs, there was virtually no effective way to deliver drugs based on proteins or peptides. By incorporating drugs with protein or peptide structures into SLNs, it has become possible to increase their stability and delivery efficiency to the target site. Therapeutically important polypeptides incorporated into SLN carriers are calcitonin, cyclosporin A, insulin, LHRH, and somatostatin. In contrast, HBs (hepatitis B) and malaria antigens are important protein antigens. The model protein drugs delivered with SLN are bovine serum albumin and lysozyme [106]. It has been reported that SLNs are effective carriers of doxorubicin and tobramycin into the brain. Importantly, SLNs carrying these drugs are capable of crossing the blood-brain barrier [107,108,109,110], a physical and enzymatic barrier between blood vessels and neural tissue. The barrier protects the central nervous system from pathogens and controls the selective transport of certain molecules from blood to cerebrospinal fluid [111,112]. The use of pure drugs for therapies that require passage across this barrier, without support from a carrier, is ineffective, and drugs encapsulated in SLNs are able to do this. SLNs are used as carriers for chemotherapeutic substances that would otherwise have very little delivery to cancer cells [113]. The low solubility of drugs in water, their poor bioavailability, and low therapeutic index (tells how many times the lethal dose is greater than the effective dose) determine their low efficacy. Furthermore, increased resistance to cytostatics also contributes to the ineffectiveness of classical chemotherapy [114,115]. Due to the attachment of specific ligands to SLN-type carriers, it is possible for them to bind directly to the appropriate receptor located in tumor cells and simultaneously release the active substance into the tumor cell [116]. SLNs used as carriers of therapeutic substances provide an opportunity to increase the effectiveness of therapy [113].

#### 5.4.2. Nanostructured Lipid Carriers (NLCs)

Nanostructured lipid carriers (NLCs) are capable of delivering drugs in a number of ways: topical application, oral application, in the lungs, eyes, and brain [117].

In dermatology, topical application, directly to the skin lesions, is the preferred mode of application because it mitigates side effects that can occur after oral and parenteral administration of drugs. In addition, the high concentration of the drug is maintained at the desired site for a long time. A major challenge for researchers was the low permeability of the drug through the stratum corneum, which is a barrier to toxins, molecules, and therapeutic substances. In dermatological applications, NLC offers the opportunity to be used in conventional creams and emulsions to provide controlled release and protection of the active ingredient and to minimize the risk of skin irritation. When drugs combined with NLC were used, an increase in their release rate was observed, regardless of the introduction of different emulsifiers during their synthesis [117]. It has been confirmed that the use of drugs encapsulated in NLCs provides a longer release time of the active ingredient and the whole system is not toxic under different therapeutic conditions. Examples of drugs that have been used in an encapsulated form in NLC are podophyllotoxin [118] and anti-inflammatory drugs such as flurbiprofen [119], indomethacin [120], celecoxib [116], N-palmitoyl-ethanolamine [121], etc.

Oral administration is the easiest way to administer a drug because it is painless and allows accurate dosage control. However, many drugs are poorly soluble and poorly bioavailable after oral administration due to various chemical, physical, and enzymatic barriers in the gastrointestinal tract. New drug delivery systems have been developed to overcome limitations, increase therapeutic efficacy (which would allow dose reduction), and mitigate side effects. The use of NLC increases the solubility of the active ingredient and enhances its permeability and bioavailability by protecting the drug from physiological pH and enzymatic degradation [122,123]. In the gastrointestinal tract, lipids from NLCs are partially digested in the stomach and then degraded in the small intestine to diacylglycerols and free fatty acids. Due to the presence of lipids, drugs encapsulated in NLCs remain longer in the stomach and colon, increasing drug absorption. NLCs stimulate bile secretion, which facilitates micelle formation [124].

A very promising and noninvasive route of drug delivery is inhalation through the lungs. It can be used for both local and systemic effects. The advantage of this route of drug delivery is the rapid absorption associated with the large lung surface area (nearly 100 m^2^) and the slow metabolism of the drug due to low enzymatic activity. It has proved promising in the treatment of cancer, metabolic problems, diabetes, chronic pain, infections, and autoimmune diseases [125,126]. In this route of administration, NLC provides:

- protection from degradation;
- homogeneous distribution of the drug in the alveoli;
- prolonged release time of the active substance;
- increased solubility of the active ingredient;
- reduced/mitigated side effects [127].

Barriers to the effectiveness of inhaled drugs include the geometry of the airways, moisture, and the presence of macrophages in the alveoli. The efficacy of the drug also depends on the size of the NLC, the homogeneous distribution of the drug in the lungs, its dose, the precipitation of the site, and the type of disease [128]. NLCs, like other inhaled formulations, must be nontoxic, biocompatible with the human body, nonirritating, and have an appropriate pH (3.0–8.5) [2].

Due to its unique anatomy and physiology, the eye is one of the best-protected organs in the human body. Drug delivery to this organ, especially to its posterior part, is very difficult due to the presence of various barriers: cornea, sclera and retina, blood-water, and blood-retina. Topical administration is non-invasive and very effective, especially in the anterior part of the eye. However, drug bioavailability is low, mainly due to the short residence time in the target tissue and the presence of anatomical barriers. The penetration of active substances into the posterior part of the eye is less than 5%; therefore, other more invasive routes of drug administration are used, such as injections into the vitreous body and subconjunctiva, but they can cause infection, bleeding, and even loss of vision [129,130]. Drugs that have been successfully injected into the back of the eye include triamcinolone acetonide, mangiferin, voriconazole, cyclosporine A, or tobramycin [131].

The incidence of central nervous system diseases, such as Alzheimer’s and Parkinson’s disease, strokes, brain cancer, epilepsy, and bran infections, has increased significantly in recent decades. Their effective treatment is challenging, as almost 98% of the proposed drugs cannot cross the blood-brain barrier [83]. NLCs have been shown to be effective in delivering drugs to the brain because their retention in brain blood vessels was much higher than that of pure drugs. NLC surface functionalization with polysorbate 80, polyethylene glycol (PEG), low-density ligands (transferrin, lactoferrin, lipoprotein), low-density lipoprotein (LDL), and OX26 antibodies facilitate passage through the blood-brain barrier and delivery of the drug to the brain [132]. NLCs are preferred to SLNs due to their smaller size and potential for higher drug loading, although there has been no sufficient evidence to support the efficacy of the former [83].

Recently, it has been demonstrated that methotrexate encapsulated in NLC can be introduced intradermally into pathologically affected joints [133]. The efficacy of methotrexate incorporated into NLC is evidenced by reduced levels of various inflammatory markers and bone degrading enzymes. Another drug administered in NLC is flurbiprofen, which is used to treat inflammatory joint conditions. However, due to its short half-life in the elimination phase, it had to be used for a longer period of time. Flurbiprofen is encapsulated in NLCs to evaluate the potential for intradermal delivery and diffusion into epidermal hair follicles [134].

The major drugs used in NLC delivery systems, along with their applications, are presented in Table 6.

## 6. Lipid Nanoparticles as Carriers of Viral Proteins

The emergence of a previously unknown SARS-CoV-2 virus (COVID-19) in Hubei Province, China, in December 2019 shocked the world. The virus has spread rapidly, taking over the entire world [156,157]. The World Health Organization (WHO) declared it a public health emergency of international concern [158]. Scientists from all over the world have unanimously agreed that a vaccine against COVID-19 is the most effective way to combat the pandemic and protect human health. Consequently, researchers around the world pursued the development of a vaccine against this virus [159].

Thanks to the efforts of researchers and international collaborations, a number of potential vaccines capable of combating COVID-19 have been rapidly developed. We can divide them into the following:

- vector vaccines based on fragments of other viruses,
- mRNA vaccines;
- subunit vaccines, which contain purified viral proteins in their composition.

Vector-based vaccines are based on cold viruses, which are harmless to humans and are found in chimpanzees. These viruses, which are part of the adenovirus family, have been modified to contain the gene responsible for the production of the SARS-CoV-2 entry protein [160,161]. Research on mRNA-based vaccines has been conducted for a long time. Their main principle is to encode the antigen in mRNA and then deliver the transcript to the cytoplasm of the host cell using a non-viral delivery system that enables antigen expression and induction of an antigen-specific immune response [162]. Currently, there are three main types of RNA-based vaccines:

- conventional, non-amplifying base-modified messenger RNA (mRNA) molecules;
- non-amplifying mRNA (bmRNA) that contain chemically modified nucleotides;
- self-amplifying mRNAs (saRNAs or replicons) that retain auto replicative activity, extracted from a viral RNA vector [160,162].

The COVID-19 vaccine contains an mRNA molecule that contains the instructions for making the spike protein. This protein is found on the surface of the SARS-CoV-2 virus. The virus needs it in order to enter the body’s cells. When a person is given a vaccine, some of their cells read the mRNA instructions and temporarily produce the “spike” protein. The vaccinated person’s immune system recognizes this protein as foreign, produces antibodies, and activates T lymphocytes whose purpose is to fight the virus. If the vaccinated person later comes into contact with the SARS-CoV-2 virus, their immune system will recognize it and be ready to defend the body; the mRNA from the vaccine does not remain in the body but breaks down soon after vaccination [163].

Scientists are constantly investigating the possibility of efficiently and rapidly transferring the therapeutic substance into the human body; moreover, the question of whether lipid nanoparticles can be carriers of viruses was born. By using NLPs as mRNA carriers, it has become possible to effectively and safely control the SARS-CoV-2 virus. Efficient delivery of these vaccines requires that the mRNA has to be protected against extracellular degradation [164]. One of the first vaccines based on mRNA and lipid nanoparticles was Moderna’s non-replicating mRNA vaccine, mRNA-1273 [165,166,167], while BioNTech/Pfizer’s vaccine, BNT162b2, was the first to be circulated in the UK and then in Canada, with an impressive efficacy of 95% [168]. The most important and effective vaccines developed based on RNA and lipid nanoparticles are summarized in Table 7 [162].

Nanoparticles and viruses operate on a similar size scale so that nanoparticles can enter cells to enable the expression of antigens from delivered nucleic acids (mRNA and DNA vaccines). Many vaccine technologies use such properties to encapsulate genomic material or protein/polypeptide (oligopeptide) antigens in lipid nanoparticles (LNP) [169]. Both BioNTech/Pfizer and Moderna use lipid nanoparticles as mRNA carriers in their vaccines (Table 8).

## 7. Summary

This literature review describes the properties and applications of nanocarriers such as liposomes, niosomes, and lipid nanoparticles. Active substance carriers are required to be effective and safe. An important part of the treatment is the delivery of the active substance to a specific site in the body and its release for the desired time. Amongst the various types of drug nanocarriers, lipid nanoparticles have shown great promise in the efficient delivery of therapeutics by various routes of administration. The excipients used for the fabrication of lipid nanoparticles are biocompatible, biodegradable, non-irritating, and most of them are approved with GRAS status. The main task of lipid nanoparticles is to increase the bioavailability of the incorporated active ingredient, to control therapeutics release, and improve intracellular permeation and regulation of active compounds delivery to well-defined sites. In addition, these materials can act as effective carriers of anticancer compounds, the use of which will minimize the occurrence of treatment side effects. Accordingly, they can be used in COVID-19 therapy and prevention.

## Figures and Tables

**Table 1:** The most common methods for producing liposomes.

Type of Method	Description of the Method	Ref.
**MECHANICAL DISPERSION METHOD**		
**Sonication**	Sonication is the best-known and widespread method for the synthesis of liposomes, especially small monolayer vesicles (SUV). Its main disadvantages are very low encapsulation efficiency, elimination of large molecules, metal contamination from the probe tip, possible degradation of phospholipids, and presence of MLV. There are two sonication techniques: (a) probe-tip sonication—the energy needed for lipid dispersion is very high. Accumulation of energy at the tip of the sonicator probe can cause a local rise in temperature. Therefore, the reaction vessel must be immersed in a water or ice bath to control the temperature. During sonication (up to 1 h), more than 5% of the lipids can deesterify. In addition, the use of a titanium-coated probe causes contamination of the solution. (b) sonication using a bath—liposome dispersion is placed in a bath-type sonicator. Controlling the temperature of the dispersion is much easier in this method than in the sonication using a probe. The sonicated liposomes can be secured in a sterile vessel other than the probe or in an inert atmosphere.	[12,22]
**French pressure cell: extrusion**	The method involves injecting and pushing MLV vesicles through a small hole. It is more advantageous than the sonication method because the resulting liposomes are larger in size and resemble encapsulated substances in form. The production of liposomes requires high temperatures and a limited working volume.	[22,23]
**Freeze-thawed liposomes**	This method involves freezing the SUV liposomes for a short period of time and then leaving them to thaw for a long time. This technique leads to the dispersion of lipids and the formation of large monolayer vesicles (LUVs). Its greatest limitation is the increase in the phospholipid concentration and ionic strength of the medium.	[22]
**High-pressure homogenization**	The method is mainly used to obtain small vesicles (SUV) and to reduce the size of multilamellar liposomes.	[24]
**Membrane extrusion**	A common industrial method for obtaining liposomes and consists of extruding the emulsion through a polycarbonate membrane with a uniform pore size (N × 10,000 Daltons). Lipids are pressed through the membrane, and then they are repeatedly circulated for a specific time, pressure, temperature, and flow rate. This technique requires less energy and fewer mechanical systems (which can cause additional fouling).	[24]
**SOLVENT DISPERSION METHOD**		
**Ether injection (solvent vaporization)**	Consists of the preparation of solutions of lipids dissolved in diethyl ether or ether-methanol mixture, which are then gradually injected into an aqueous solution of the material, which is encapsulated at 55 to 65 °C or under reduced pressure. Systematic removal of the ether under vacuum leads to liposomes. The main disadvantage of this method is the heterogeneous particle size, ranging from 70 to 200 nm. Compounds that are encapsulated are also exposed to organic solvents at high temperatures.	[12]
**Ethanol injection**	In this method, a lipid ethanol solution is rapidly injected into an excess of 0.16 M KCl, resulting in the immediate preparation of MLV-type liposomes. A significant disadvantage of this method is the heterogeneous size of the particles obtained (30 to 110 nm), very high dilution of the liposomes, and the need to remove ethanol.	[12]
**Reverse-phase evaporation method**	The technique allows the production of liposomes with a high water to lipid volume ratio and allows the retention of a significant percentage of aqueous material. It involves the formation of inverted micelles that are shaped by sonication of a mixture of a buffered aqueous phase containing water-soluble molecules (which will be encapsulated in liposomes) and an organic phase (in which amphiphilic molecules are dissolved). In the next step, the organic solvent is removed, and thus the inverted micelles are transformed into a viscous state and gel form. Excess phospholipids in the environment ensure the formation of a complete bilayer around the residual micelles, which results in the formation of liposomes. Liposomes produced by this method can be made from multiple lipid preparations and have a lipid-to-water volume ratio four times higher than that of multilamellar liposomes.	[22,25]
**DETERGENT REMOVAL METHOD (REMOVAL OF UNENCAPSULATED MATERIAL)**		
**Dialysis**	Detergents are used to solubilize the lipids at their critical micellization concentrations (CMC). These detergents are then removed by dialysis, which can be performed in dialysis bags immersed in buffer solutions that do not contain detergents. While the detergent is removed, the micelles easily bind to the phospholipids and form the LUV structure.	[12]
**Detergent removal of mixed micelles**	Detergent absorption is possible by shaking the mixed micelle solution with organic polystyrene absorbents. A major advantage of using these types of absorbents is that they can eliminate detergents with very low CMC.	[12]

**Table 2:** Compounds used to obtain niosomes.

	Type of Compounds	Examples	Ref.
**Nonionic surfactant**	Alkyl ethers	Alkyl glycerol ether, Hexadecyl diglycerol ether	[34]
	Crown ethers	Alpha, omega-hexadecyl-bis(1-aza-18-crown-6)(Bola)	[35,36]
	Alkyl esters	Span 20, Span 40, Span 60, Span 80, Span 65, Span 85	[37,38,39,40,41,42,43]
		Tween 20, Tween 40, Tween 60, Tween 80, Tween 65, Tween 85	
	Fatty acids	Stearic acid, palmitic acid	[44]
	Fatty alcohols	Stearyl alcohol, cetyl alcohol	
	Block copolymers	Pluronic L64, Pluronic 105	[45,46]
**Lipids**	Steroids	Cholesterol	[47]
**Charged molecule**	Negative charge	Dicetyl phosphate, phosphatidic acid, lipoamino acid	[48,49]
	Positive charge	Stearylamine, stearyl pyridinium chloride, cetylpyridinium chloride	

Type of Compounds

Examples

Ref.

**Table 3:** Methods for obtaining niosomes.

Type of Method	Description of the Method	Ref.
**Thin-Film Hydration (TFH) Method**	Initially, the TFH technique was used to synthesize liposomes, but over time, it was also used to obtain niosomes. In this method, surfactants and cholesterol are homogeneously dissolved in an organic solvent such as chloroform or a mixture of solvents. The solvent is completely evaporated using a rotary vacuum evaporator, resulting in a thin film on the inner surface of the flask. The resulting film is rehydrated using either water alone or phosphate-buffered saline (PBS), which usually contains the encapsulation drug. Once the rehydration is complete, MLV niosomes of various diameters are formed.	[33,55]
**Solvent Injection (SI) Method**	The SI technique involves dissolving the surfactant and cholesterol with diethyl ether or ethanol. The homogeneous solution is placed in a syringe pump and introduced dropwise through a needle into an aqueous solution at a constant temperature (which is higher than the boiling point of the organic solvent). The residual solvent is removed by evaporation in a vacuum rotary evaporator. This process leads to monolayer vesicular niosomes of various sizes.	[33]
**Reverse Phase Evaporation (REV) Method**	The REV method was first presented by Szoka and Papahadjopoulos [26] in 1978 as a technique to obtain LUV-type niosomes. It involves the preparation of two phases, organic and aqueous. The organic phase usually consists of a mixture of ether and chloroform, containing Span 60, cholesterol, and stearoylamine to form the membrane. The aqueous phase consists of water or PBS in which the drug is dissolved. The organic phase is mixed with the aqueous phase, and then the whole mixture is vigorously shaken or subjected to ultrasound to obtain the emulsion. The next step of the process is to evaporate the organic phase using a constant temperature vacuum evaporator, resulting in LUV-type niosomes.	[26,33]
**The Bubble Method**	Niosomes are obtained without the use of organic solvents. Surfactants, additives (e.g., cholesterol), and the buffer (pH 7.4) are added into a three-neck round-bottom flask. The flask is placed in a water bath to control the temperature. The dispersion of surfactants and additives occurs at 70 °C. To obtain a homogeneous dispersion, a high-speed homogenizer is first used, stirred for 15–30 s, and then bubbled with a 70 °C nitrogen gas. Nitrogen gas is passed through a sample of homogenized surfactants resulting in the formation of large unilamellar vesicles.	[56,57,58]
**Freeze and Thaw Method**	It is an improved and much simpler method of niosome preparation derived from the TFH technique. MLV-type niosome suspension prepared by the TFH method is frozen in liquid nitrogen and then thawed several times in a short time using a water bath.	[59]
**Dehydration–Rehydration Vesicles (DRV) Method**	It was first described by Kirby and Gregoriadis [34]. SUV-type niosomes prepared by the TFH technique are used to form MLVs. Small monolayer vesicles (SUVs) are separated by centrifugation, and then SUV-type niosomes containing the drug are added to the aqueous phase. The resulting suspension was lyophilized overnight. After the dried product was rehydrated, multilamellared niosomes formed.	[33]
**Supercritical Carbon Dioxide Fluid (scCO2) Method**	It is a new method to obtain niosomes, and it was first presented by Manosroi et al. [60]. Surfactant, cholesterol, PBS with glucose and ethanol are placed in a glass cell of a constant volume and two windows. CO_2_ is then introduced into the cell while maintaining a pressure of 200 bar and a temperature of 60 °C. Niosomes are obtained after 30 min of stirring all ingredients on a magnetic stirrer and then lowering the pressure. With this method, LUV-type niosomes with sizes ranging from 100 to 440 nm can be obtained. The greatest advantage of this method is the one-step process, which does not require the use of toxic, flammable, and volatile organic solvents.	[33,60]
**Heating Method (HM)**	The technique has been recently developed and is used to obtain nanocarriers, including niosomes. The method involves the addition of surfactants, cholesterol, and active ingredients to an aqueous phase (such as PBS). The solution is prepared by stirring and heating the aqueous phase, and then a 3% *w*/*v* (weight/volume) polyol such as glycerol is added to the resulting solution. The main advantage of the method is that there is no need for toxic and volatile organic solvents.	[33]

**Table 4:** Selected lipids used for LNP synthesis and their structure.

Type of Lipid	Structure
**Monoacylglycerols**	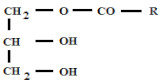
**Diacylglycerols**	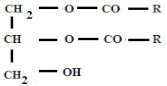
**Triacylglycerols**	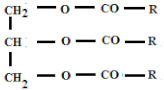
**Fatty acids**	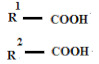

where, R, R^1^, R^2^ are alkyl chains.

**Table 5:** Pros and cons of different lipid nanoparticles production.

Methods	Advantages	Disadvantages
**Hot HPH**	versatile, avoid organic solvent, easy scalability, and short production time	high temperature can cause degradation, conformational changes in protein, changes in particle size (coalescence of particles), burst release due to high emulsifiers concentration
**Cold HPH**	avoid thermal exposure of the drug; good for temperature-labile drugs or hydrophilic drugs	higher Polydispersity index (PDI)
**Microemulsion**	no need for specialized equipment (robust) and low energy for production	high concentrations of surfactants and co-surfactants, presence of large amounts of water in the system
**Emulsification-solvent evaporation**	avoid heat during production thus useful for thermolabile drugs; simple procedure	solvent residues
**Emulsification/ultrasound/sonication**	no organic solvent residue, no burst release, high lipid concentration, versatile, avoid use of organic solvent, better drug loading than HPH	metallic particle contamination, broader particle size (higher PDI)
**Emulsification-solvent diffusion**	simple procedure, fast drug release (drawback when slow release is required)	low lipid content, low EE and DL, organic solvent residue, lack of scale-up
**Membrane Contactor**	simple method, control of particle size by selection of process parameters (size of membrane pores)	limited scaling up possibility

**Table 6:** Drugs used in the NLC delivery system and their applications.

Drug	Solid/Liquid Lipids	Use	Ref.
**Anticancer Drugs**			
**5-fluorouracil and retinoic acid conjugate**	Lecithin/oleic acid	Large intestine cancer in people	[135]
**Oridonin**	GMS/MCT	Extension of mean lifetime of retention of oridonin encapsulated in NLC coated with PEG	[136]
**Docetaxel**	GMS/oleic acid	In vivo studies have shown greater effectiveness in treatment of malignant melanoma and weaker side effects	[137]
**Etoposide**	GMS/soybean oil	Etoposide in NLC was optimized formulation of enhanced bioavailability in oral administration of the system, showing high cytotoxicity towards plano-epithelial cells of lung cancer	[138]
**Topotecan**	Stearic acid/oleic acid	Incorporation of topotecan to NLC improved its properties, chemical stability and increased its cytotoxicity	[139]
**Anti-inflammatory drugs**			
**Flurbiprofen**	GMS/MCT	Flurbiprofen encapsulated in NLC showed better permeability on topical application and was more effective than in oral administration	[119]
**Ketoprofen**	Compritol 888 ATO/labrafac lipophile	The new system of drug entrapped in cyclodextrin (inclusion compound) and encapsulated in NLC improved the therapeutic effectiveness of ketoprofen and the safety of its use	[140]
**Quercetin**	GMS/MCT	Studies of penetration of quercetin through the skin in vitro and in vivo proved that its encapsulation in NLC increased its retention in the epidermis and thus improved the therapeutic effect.	[141]
**Celecoxib**	Precirol/squalene	Pharmacodynamic studies of celecoxib in NLC showed extended to 24 h activity of the drug	[116]
**Antifungal drugs**			
**Silybin**	GMS/MCT	The drug in GMS/MCT reached the blood circulation system faster but was to a high degree captured by RES (reticular endothelial system) in the organs (in particular in the liver)	[142]
**Fluconazole**	Compritol 888 ATO (CA)/oleic acid	Encapsulation of the drug in NLC ensured effective and extended release of the drug in the skin, which increased effectiveness of fungal infection	[143]
**Itraconazole**	GMS, prekirol/oleic acid, miglyol	Itraconazole was encapsulated to stable NLC whose properties were unaffected on introduction to the lungs	[144]
**Glucocorticoids**			
**Dexamethasone**	GMS/medium chain triacyclglycerols (MCT)	Dexamethasone in a complex incorporated into NLC showed high stability and extended release of active ingredient	[145]
**Fluticasone**	Precirol ATO 5 /labrasol	The presence of PEG-containing lipids in NLC improved the drug stability	[146]
**Corticosteroids**			
**Triamcilone acetonide**	Prekirol ATO 5 /squalene	In vivo tests in mice proved increased stability of the drug encapsulated in NLC and the possibility of its delivery to the back part of the eye	[147]
**Antioxidants**			
**Resveratrol**	Compritol 888 ATO/miglyol	Resveratrol encapsulated in NLC showed higher antioxidation activity than the same drug incorporated in SLN	[148]
**Curcumin**	GMS/MCT	The encapsulation of curcumin in an NLC resulted in an increase in its antimalarial activity	[149]
**Lutein**	Precirol ATO 5	NLC was shown to protect lutein against the simulated gastric fluid and allowed its slow release in the simulated intestinal fluid	[150]
**Immunosuppressants**			
**Tacrolimus**	GMS/oleic acid	Tests in vitro revealed that encapsulation of tacrolimus in NLC increases its penetration coefficient to a value of 1.64 times greater than that of the commercial ointment with tacrolimus Protopic^®^	[151]
**Antipsoriatic drugs**			
**Acitretin**	Precirol ATO 5 /oleic acid	The system Precirol ATO5/ oleic acid / NLC proved to be effective in treatment of psoriasis	[152]
**Psoralen**	Precirol/squalene	The system Precirol/ squalene showed increased penetration and possibility of controlled drug release	[117]
**Anesthetic drugs**			
**Lidocaine**	Compritol 888 ATO/miglyol 810	Topical application of lidocaine encapsulated in NLC in gel form permits slow development of local anesthesia in guinea pigs	[153]
**Antimalarial drugs**			
**Dihydroartemisinin**	GMS/miglyol 812	The course of release of dihydroartemisinin incorporated in NLC indicated the formation of diphase pattern in the beginning phase and extended release later	[154]
**Sunscreens**			
**Oxybenzone**	GMS/miglyol	Incorporation of oxybenzone to NLC has considerably increased its sunscreen index	[155]

**Compritol 888 ATO**—glyceryl dibehenate; **MCT**—medium chain triacylglycerols; **GMS**—acylglycerol monostearate; **PEG**—polyethylene glycol.

**Table 7:** Summary of mRNA-based vaccines against the SARS-CoV-2 virus (modified from [162]; original paper under the terms of the Creative Commons CC BY-NC 3.0).

Company	Type of mRNA	Delivery System
**Moderna**	bmRNA	LNP
**BioNTech/Pfizer**	bmRNA	LNP
**ICL**	saRNA	LNP
**Arcturus**	saRNA	LNP
**CureVac**	mRNA	LNP

**Table 8:** Comparison of BioNTech/Pfizer and Moderna vaccines based on mRNA and lipid nanoparticles.

	BioNTech/Pfizer	Moderna
**Name of the medicinal product**	Comirnaty concentrate for dispersion for injection. COVID-19 mRNA vaccine (with modified nucleosides)	COVID-19 Vaccine Moderna dispersion for injection. COVID-19 mRNA vaccine (with modified nucleosides)
**List of excipients**	lipids: ((4-hydroxybutyl)azanediyl)bis(hexane-6,1-diyl)bis(hexyl 2-decanoate), (ALC-0315) 2-[(polyethylene glycol)-2000]-*N*,*N*-ditetradecylacetamide, (ALC-0159) 1,2-distearoyl-sn-glycero-3-phosphocholine (DSPC) cholesterol potassium chloride potassium dihydrogen phosphate sodium chloride disodium phosphate dihydrate sucrose water for injection	lipid SM-102 cholesterol 1,2-distearyl-sn-glycero-3-phosphocholine (DSPC) 1,2-dimethyl-rac-glycero-3-methoxypolyethylene glycol, molecular weight 2000 (PEG2000 DMG) trometamol trometamol hydrochloride acetic acid sodium acetate trihydrate sucrose water for injection
**Ref.**	[170]	[171]

## Data Availability

Not applicable.
